# Supplementary material for: Gene Expression Profiling of Skeletal Muscles
Source: Genes (Basel). 2021 Oct 28;12(11):1718. doi: 10.3390/genes12111718 (PMC8621074; doi:10.3390/genes12111718)
Supplement: Supplementary file 1 [file genes-12-01718-s001.zip › genes-1413573-supplementary.pdf]

Gene expression profiling of skeletal muscles

Supporting information

Sarah I. Alto[1,2], Chih-Ning Chang[1,2], Kevin Brown[1,3], Chrissa Kioussi[1,2,*], Theresa M. Filtz[1,2]

[1]Department of Pharmaceutical Sciences, College of Pharmacy, Oregon State University Corvallis, Oregon, United States of America

[2]Molecular and Cellular Biology Graduate Program, Graduate School, Oregon State University, Corvallis, Oregon, United States of America

[3]School of Chemical, Biological, and Environmental Engineering, College of Engineering, Oregon State University, Corvallis, Oregon, United States of America

[*]Corresponding author:

Email: chrissa.kioussi@oregonstate.edu (CK); TEL 541-737-2179

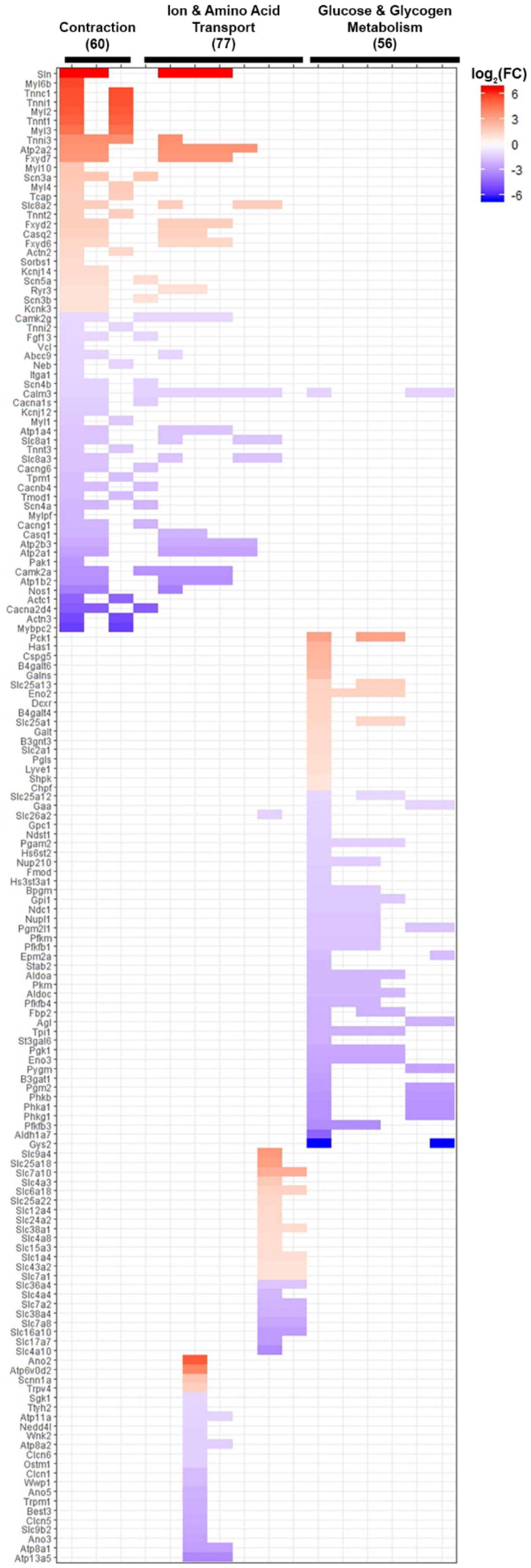
Contraction
(60)
Ion & Amino Acid
Transport
(77)
Glucose & Glycogen
Metabolism
(56)
log2(FC)
6
3
0
-3
-6
Sln
Myl6b
Tnnc1
Tnni1
Myl2
Tnnt1
Myl3
Tnni3
Atp2a2
Fxyd7
Myl10
Scn3a
Myl4
Tcap
Slc8a2
Tnnt2
Fxyd2
Casq2
Fxyd6
Actn2
Sorbs1
Kcnj14
Scn5a
Ryr3
Scn3b
Kcnk3
Camk2g
Tnni2
Fgf13
Vcl
Abcc9
Neb
Itga1
Scn4b
Calm3
Cacna1s
Kcnj12
Myl1
Atp1a4
Slc8a1
Tnnt3
Slc8a3
Cacng6
Tpm1
Cacnb4
Tmod1
Scn4a
Mylpf
Cacng1
Casq1
Atp2b3
Atp2a1
Pak1
Camk2a
Atp1b2
Nos1
Actc1
Cacna2d4
Actn3
Mybpc2
Pck1
Has1
Cspg5
B4galt6
Galns
Slc25a13
Eno2
Dcxr
B4galt4
Slc25a1
Galt
B3gnt3
Slc2a1
Pgls
Lyve1
Shpk
Chpf
Slc25a12
Gaa
Slc26a2
Gpc1
Ndst1
Pgam2
Hs6st2
Nup210
Fmod
Hs3st3a1
Bpgm
Gpi1
Ndc1
Nupl1
Pgm2l1
Pfkm
Pfkfb1
Epm2a
Stab2
Aldoa
Pkm
Aldoc
Pfkfb4
Fbp2
Agl
Tpi1
St3gal6
Pgk1
Eno3
Pygm
B3gat1
Pgm2
Phkb
Phka1
Phkg1
Pfkfb3
Aldh1a7
Gys2
Slc9a4
Slc25a18
Slc7a10
Slc4a3
Slc6a18
Slc25a22
Slc12a4
Slc24a2
Slc38a1
Slc4a8
Slc15a3
Slc1a4
Slc43a2
Slc7a1
Slc36a4
Slc4a4
Slc7a2
Slc38a4
Slc7a8
Slc16a10
Slc17a7
Slc4a10
Ano2
Atp6v0d2
Scnn1a
Trpv4
Sgk1
Ttyh2
Atp11a
Nedd4l
Wnk2
Atp8a2
Clcn6
Ostm1
Clcn1
Wwp1
Ano5
Trpm1
Best3
Clcn5
Slc9b2
Ano3
Atp8a1
Atp13a5

Figure S1. Evaluation of Reactome overrepresented pathway analysis. The statistically significant DE genes were filtered into curated molecular pathways using the *ReactomePA* R package. The Reactome overrepresented pathway analysis tool uses a list of genes and filters the genes into curated molecular pathways. Specific molecular pathways were over-represented based on the input genes exceeding the proportion of genes randomly expected for a particular molecular pathway. Genes with increased expression in So or Ta are labeled in red or blue, respectively. The heat map indicated the log_2(FC) of 160 DE genes associated with the 16 overrepresented pathways identified by the Reactome database before filtering based on the absolute FC being greater than or equal to two. Based on description similarities, the sixteen pathways were combined into three major categories: Contraction, Ion & Amino Acid Transport, and Glucose & Glycogen Metabolism.

Table S1. Differentially Expressed Genes in So and Ta involved in Ion Transport and Glycosaminoglycan biosynthesis pathways.

Gene Symbol	Gene Name	Fold Change (So/Ta)	Localization and Biochemical Properties
**Amino Acid and Ion Transport**			
**P-type 2A ATPases or Sarco-(endo)-plasmic reticulum calcium pumps (SERCA)**			
Atp2a2	ATPase, Ca^{2+} transporting, cardiac muscle, slow twitch 2	13.1	regulates Ca^{2+} homeostasis and signaling in slow-twitch skeletal myofibers [1]
Atp2a1	ATPase, Ca^{++} transporting, cardiac muscle, fast-twitch 1	-6.1	regulates Ca^{2+} homeostasis and signaling in fast-twitch skeletal myofibers [2]
**P-type 2B ATPases or Plasma Membrane Ca^{2+}-ATPases (PMCA)**			
Atp2b3	ATPase, Ca^{++} transporting, plasma membrane 3	-4.9	transports calcium ions [3]
**P-type 2C ATPases or Sodium-Potassium Pumps**			
Fxyd7	FXYD domain-containing ion transport regulator 7	12	decreases intrinsic K+ affinity without extracellular Na+ competition or effect on membrane potential [4]
Fxyd2	FXYD domain-containing ion transport regulator 2	3.3	membrane potential control based on changing Na+ and K+ affinities [4]
Atp1b2	ATPase, Na^+/K^+ transporting, beta 2 polypeptide	-8.3	phosphorylation and ion transport [5]
Atp1a4	ATPase, Na^+/K^+ transporting, alpha 4 polypeptide	-2.9	phosphorylation and ion transport [6]
**P-type 4 ATPases or Phospholipid Flippases**			
Atp8a1	ATPase, amino-phospholipid transporter (APLT), class I, type 8A, member 1	-6.3	mediates amino-phospholipids translocation; dependent on phosphatidylserine for ATPase activity [7]
**P-type 5 ATPases or Orphan Transporters**			
Atp13a5	ATPase type 13A5	-10	Transports unknown ion in the brain and stomach [8]
**V-type ATPase or Plasma membrane H^+-ATPases**			
Atp6v0d2	ATPase, H^+ transporting, lysosomal V0 subunit D2	17.4	couples proton transport and ATP hydrolysis
**Calcium-activated Chloride Channels**			

Ano2[#]	anoctamin 2	39.7	involved in membrane potential of presynaptic membranes in photoreceptors and olfactory sensory neurons [9]
Ano3	anoctamin 3	-5.8	involved in intracellular calcium-activated anion current [10]
Best3	bestrophin 3	-4.7	regulates calcium release from intracellular calcium storage [11]
Ano5	anoctamin 5	-4.5	involved in sarcolemmal repair and myoblast fusion in skeletal muscle [12]
**Voltage-sensitive Chloride Channels**			
Clcn5	chloride channel, voltage-sensitive 5	-5.1	Involved in endosomal acidification in skeletal muscles [13]
Clcn1	chloride channel, voltage-sensitive 1	-3.5	involved in membrane potential in skeletal muscles [14]
**Calcium Channels**			
Cacna2d4[#]	calcium channel, voltage-dependent, alpha 2/delta subunit 4	-23.6	regulates gating & ligand binding and increases in current amplitude [15]
Cacng1	calcium channel, voltage-dependent, gamma subunit 1	-4.1	decreases Ca^{2+} entry during stimulation of skeletal muscle [16]
Cacnb4	calcium channel, voltage-dependent, beta 4 subunit	-3.4	increases current amplitude and regulates activation and inactivation kinetics [17]
Cacng6	calcium channel, voltage-dependent, gamma subunit 6	-3.2	reduces the current amplitude [18]
**Solute Carrier Family**			
Slc22a2	solute carrier family 22 (organic cation transporter), member 2	80.77	removes noxious cationic compounds [19]
Slc9a4	solute carrier family 9 (sodium/hydrogen exchanger), member 4	11.5	involved in cell size and not intracellular pH [20]
Slc25a18	solute carrier family 25 (mitochondrial carrier), member 18	9.9	transports metabolites across inner mitochondrial membrane [21]
Slc7a10	solute carrier family 7 (cationic amino acid transporter, y+ system), member 10	7.1	transports for small neutral amino acids, especially D-serine and glycine
Slc4a3	solute carrier family 4 (anion exchanger), member 3	3.7	lowers the CO_2 load with the muscle cell [22]
Slc8a2	solute carrier family 8 (sodium/calcium exchanger), member 2	3.4	intracellular calcium concentration regulation; synaptic plasticity [23]

Slc6a18	solute carrier family 6 (neurotransmitter transporter), member 18	3.1	transports neutral amino acids in the presence of sodium and chloride ions
Slc25a13	solute carrier family 25 (mitochondrial carrier, adenine nucleotide translocator), member 13	3.1	involved a mitochondrial aspartate glutamate carrier and the urea cycle [24]
Slc4a10	solute carrier family 4, sodium bicarbonate cotransporter-like, member 10	-9.3	involved in regulating neuronal pH and excitability [25]
Slc17a7 (VGLUT1)	solute carrier family 17 (sodium-dependent inorganic phosphate cotransporter), member 7	-6.8	transports glutamate and depends on electrogenic and Cl- conditions; distributed in synaptic vesicles [26]
Slc16a10	solute carrier family 16 (monocarboxylic acid transporters), member 10	-6.6	part of net efflux pathway for aromatic amino acids across the sarcolemma [27]
Slc7a8	solute carrier family 7 (cationic amino acid transporter, y+ system), member 8	-5.4	exchanges neutral amino acids [28]
Slc9b2	solute carrier family 9, subfamily B (NHA2, cation proton antiporter 2), member 2	-5.1	Part of intracellular and mitochondrial cation/proton antiport system [29]
Slc38a4	solute carrier family 38, member 4	-4.4	**UNKNOWN**
Slc7a2	solute carrier family 7 (cationic amino acid transporter, y+ system), member 2	-4.1	transports cationic amino acids correlated to nitric oxide production
Slc4a4	solute carrier family 4 (anion exchanger), member 4	-3.9	regulates intracellular pH [30]
Slc8a3	solute carrier family 8 (sodium/calcium exchanger), member 3	-3.1	controls intracellular calcium concentrations at neuromuscular junctions [31]
Slc8a1	solute carrier family 8 (sodium/calcium exchanger), member 1	-3	lowers intracellular calcium concentration during the relaxation in cardiac muscle [32,33]
**Nucleotide Sugar Transporter**			
Slc35d3	solute carrier family 35, member D3	20.07	orphan sugar nucleotide transporter; regulates platelet-dense granules [34]
**Glycosaminoglycan biosynthesis**			
Has1	hyaluronan synthase 1	6.1	induces pro-inflammation response
Cspg5	chondroitin sulfate proteoglycan 5	5.9	cell adhesion and growth, receptor binding, or cell migration
B4galt6	beta 1,4-galactosyltransferase, polypeptide 6	5.4	synthesizes lactosylceramide, a precursor of glycosphingolipids outside a cell [35]

Galns	galactosamine (N-acetyl)-6-sulfate sulfatase	4.9	breaks down keratan sulfate; present in lysosomes [36]
B3gat1	beta-1,3-glucuronyltransferase 1 (glucuronosyltransferase P)	-6.5	catalyzes biosynthesis of HNK-1 carbohydrate epitope; cell adhesion and neurite outgrowth [37]
St3gal6	ST3 beta-galactoside alpha-2,3-sialyltransferase 6	-4.5	sialylation in selectin ligand biosynthetic pathway[38]
Stab2	stabilin 2	-3.8	endocytosis of metabolic waste products, including hyaluronic acid (HA) and other modified proteins
**Amino Acid and Ion Transport Regulators**			
Sln[#]	sarcolipin	98.5	regulates sarcoplasmic reticulum Ca^{2+}- ATPases [39]
Syt6	synaptotagmin VI	25.19	involved in calcium-dependent exocytosis [40]
Adgrv1	adhesion G protein-coupled receptor V1	21.68	binds to calcium in CNS [41]
Casq2	calsequestrin 2	3.1	regulates calcium buffer in sarcoplasmic reticulum [42]
Nos1	nitric oxide synthase 1, neuronal	-12	synthesizes nitric oxide from L-arginine [43]
Camk2a	calcium/calmodulin-dependent protein kinase II alpha	-8.1	mediates second messenger effects of Ca^{2+} [44]
Casq1	calsequestrin 1	-4.2	Essential for sarcoplasmic reticulum development and Ca^{2+} storage and release [45]
**Cation Channel**			
Trpv4	transient receptor potential cation channel, subfamily V, member 4	3.2	regulates osmotic homeostasis [46]
Trpm1	transient receptor potential cation channel, subfamily M, member 1	-4.6	Proposed intracellular metabotropic glutamate receptor-coupled cation channel [47]
**Sodium Channel**			
Scnn1a	sodium channel, non-voltage-gated 1 alpha	4.4	regulates sodium reabsorption [48]
Scn3a	sodium channel, voltage-gated, type III, alpha	3.9	involved in sodium ion influx in neural tissue [49]
Scn4a	sodium channel, voltage-gated, type IV, alpha	-4	involved in sodium ion influx in skeletal muscle [50]

Positive or negative fold change represents increased gene expression in So or Ta, respectively. All references mentioned in this Table are listed in this supporting information PDF. [#]DE gene both Reactome and 20x fold change

Table S2. Differentially Expressed Genes in So and Ta organized by Immune Response, Signaling, and Cellular function pathways.

Gene Symbol	Gene Name	Fold Change (So/Ta)	Localization and Biochemical Properties
**Immune System**			
C1rb	complement component 1, r subcomponent B	206.06	part of complement system of innate immune system [51]
C1s2	complement component 1, s subcomponent 2	99.94	part of complement system of innate immune system [51]
Il27 (Ebi3 or Il-27p28)	interleukin 27	90.94	Part of specific cell-surface differentiation of T-cells and stimulate T-cells to suppress inflammation [52]
Btn2a2	butyrophilin, subfamily 2, member A2	72.97	inhibits T-cell-mediated immunity [53]
Nccrp1 (FBXO50)	non-specific cytotoxic cell receptor protein 1 homolog (zebrafish)	62.98	ubiquitous tissue expression pattern in mice [54]
Orm1	orosomucoid 1	57.38	modulates immunity, binds and carries drugs, mediates sphingolipid metabolism [55]
Klra10 (Ly49j)	killer cell lectin-like receptor subfamily A, member 10	55.78	inhibitory intracellular protein lacking a transmembrane domain and has unknown ligand specificity [56]
Pigr	polymeric immunoglobulin receptor	48.6	facilitates transcytosis of soluble immunoglobulin A and immune complexes polymeric isoforms [57]
Vsig8	V-set and immunoglobulin domain containing 8	45.89	interacts with V-Set and transmembrane signaling protein VISTA [58]
Pianp	PILR alpha associated neural protein	35.02	involved in proteolytic processing; ligand for immune inhibitory receptor Pilrα once cleaved [59]
Treml1	triggering receptor expressed on myeloid cells-like 1	29.82	dampens inflammatory response; specific to platelet and megakaryocyte alpha-granules [60]
Klrk1	killer cell lectin-like receptor subfamily K, member 1	24.36	activates natural killer T-cells and macrophages [61]
Ctla4	cytotoxic T-lymphocyte-associated protein 4	24.27	regulates T cell activation; maintains T cell homeostasis [62]
H2-Q10	histocompatibility 2, Q region locus 10	24.1	non-classical major histocompatibility complex class IB molecule that binds to inhibitory Ly49C receptor [63]
Klrd1	killer cell lectin-like receptor, subfamily D, member 1	20.29	expressed on natural killer T-cell surface; cell signaling involvement[64]
Wfdc5	WAP four-disulfide core domain 5	-50.74	pro-inflammation and protease inhibition [65]
Tlr5	toll-like receptor 5	-20.34	inflammatory response initiation [66]
**Signaling Pathways**			
Derl3	Der1-like domain family, member 3	36.55	involved in endoplasmic reticulum (ER)-associated degradation (ERAD) system [67]
Angpt4	angiopoietin 4	34.42	involved in vascular growth factor Angiopoietin signaling [68]
Rspo3	R-spondin 3	29.82	binds to cell surface receptors and activates Wnt/Beta-catenin signaling [69]

Dab1	disabled 1	28.93	binds to phospholipids and un-phosphorylated low-density lipoprotein receptor (LDLR) amino sequence [70]
Gareml (Garem2)	GRB2 associated regulator of MAPK1 subtype 2	28.86	adaptor in EGF-mediated signaling pathway [71]
**Calcium Signaling**			
Itpka	inositol 1,4,5-trisphosphate 3-kinase A	27.77	calcium signaling; facilitates phospho-group transfer in inositol phosphate metabolism [72]
Casr	calcium-sensing receptor	-33.21	maintains extracellular calcium levels [73]
Pvalb	parvalbumin	-23.46	regulates Ca^{2+} ions to sarcoplasmic reticulum to mediate relaxation in type II myofibers [74]
**Secretory Pathway involvement**			
Dmkn	dermokine	77.5	involved in wound healing [75]
Pcsk1n	proprotein convertase subtilisin/kexin type 1 inhibitor	59.69	Expressed in endocrine cells and neurons possessing a regulated secretory pathway [76]
Pmel	premelanosome protein	35.1	transmembrane glycoprotein modified in the secretory pathway [77]
**G-protein-coupled receptors**			
Olfr1033	olfactory receptor 1033	69.06	G-protein-coupled receptors (GPCR) with an unknown ligand [78]
Mrap2	melanocortin 2 receptor accessory protein 2	23.35	traffics melanocortin receptors to a cell surface [79]
**Insulin Release**			
Gipr	gastric inhibitory polypeptide receptor	38.18	stimulated insulin secretion [80]
Ghrl	ghrelin	32.57	binds to growth hormone secretagogue receptor; regulates glucose-induced insulin release [81]
Igf2bp1	insulin-like growth factor 2 mRNA binding protein 1	21.77	binds to 5′ UTR of insulin-like growth factor 2 (IGF2) mRNA and regulates its translation [82]
**Cell Motility**			
Tspan8	tetraspanin 8	-54.85	involved in protease expression, body-weight regulation in males, and cell migration [83]
**Cell proliferation**			
Tmem45b	transmembrane protein 45b	27.86	Potential cell proliferation involvement [84]
Mstn (Gdf8)	myostatin	-30.58	inhibited myoblast proliferation [85]
**Cell Apoptosis**			
Fank1	fibronectin type 3 and ankyrin repeat domains 1	32.46	regulates cell apoptosis via the AP-1 pathway [86]
**Transcription**			
Tmem233	transmembrane protein 233	-46.35	potential epigenetic control of skeletal muscle-associated genes [87]
**Protein binding, aggregation, and ubiquitination**			
Hspa1a	heat shock protein 1A	44.75	protein refolding, endocytosis, and chromosome stability [88]

Plekhd1	pleckstrin homology domain containing, family D (with coiled-coil domains) member 1	35.05	membrane binding and protein localization [89]
Btbd16	BTB (POZ) domain-containing protein 16	32.6	potential zinc-finger transcription factor [90]
Fam159b (Shisal2b)	shisa like 2B	20.26	does not target proteins for ubiquitination and degradation, unlike most Shisa proteins [91]
Hspa1b	heat shock protein 1B	20.04	mediates protein aggregation and folding; ubiquitin-proteasome pathway [88]
		**Photoreceptor Focus**	
Opn1mw	opsin 1 (cone pigments), medium-wave-sensitive (color blindness, deutan)	63.04	detects green wavelength [92]
Rp1	Retinitis pigmentosa 1	23.61	involved in photoreceptor development, organization of photoreceptor outer segments, and regulation of photoreceptor microtubules [93]
		**Gamete**	
Spats1 (Srsp1)	spermatogenesis associated, serine-rich 1	65.66	involved in establishing first male meiotic division [94]
Ces5a	carboxylesterase 5A	53.31	catalyzes carboxylic ester and water to produce an alcohol and carboxylate [95]
Meig1	meiosis expressed gene 1	41.05	involved in microtubular organelle for sperm head and flagellar formation [96]
Ell3	elongation factor RNA polymerase II-like 3	37.97	Testis-specific RNA polymerase II elongation factor [97]
Als2cr12 (Flacc1)	flagellum associated containing coiled-coil domains 1	30.37	stiffens sperm tail while allowing elastic bending [98]
Zp2	zona pellucida glycoprotein 2	29.06	involved in oocyte-sperm recognition; preventing penetration of many sperm through zona pellucida [99]
Gng13	guanine nucleotide binding protein (G protein), gamma 13	27.74	potential involvement in ovary development [100]
Ccdc169	coiled-coil domain containing 169	20.33	associate with an upstream transcription factor, SOHLH2, which is involved in differentiation during spermatogenesis and oogenesis [101]
Catsperd	cation channel sperm associated auxiliary subunit delta	-20.3	Involved in proper channel assembly and/or ion transport [102]
		**Miscellaneous**	
Csn1s2b	casein alpha s2-like B	241.61	calcium-sensitivity casein protein [103]
Gpr75	G protein-coupled receptor 75	72.96	triggers stimulation of insulin secretion[104]
Mansc4	MANSC domain containing protein 4	56.75	**UNKNOWN**
Fam216b	family with sequence similarity 216, member B	44.84	**UNKNOWN**
Selenbp2	selenium binding protein 2	39.51	binds to acetaminophen intermediate[105]
Sbk3	SH3 domain binding kinase family, member 3	37.63	**UNKNOWN**

Serpinb7	serine (or cysteine) peptidase inhibitor, clade B, member 7	28.13	**UNKNOWN**
Tmem45a2	transmembrane protein 45A2	27.42	**UNKNOWN**
Tmem151b	transmembrane protein 151B	22.96	**UNKNOWN**
Hist1h2ak	H2A clustered histone 15	21.88	one of core chromatin histones [106]
Fam227b	family with sequence similarity 227, member B	20.34	**UNKNOWN**
Tmem56 *(or Tlcd4)*	TLC domain-containing protein 4 (TRAM/Lag1/CLN8 Domain)	-25.19	**UNKNOWN**
Pld5	phospholipase D family, member 5	-21.16	**UNKNOWN**

Positive or negative fold change represents increased gene expression in So or Ta, respectively. All references mentioned in this Table are listed in this supporting information PDF. [#]DE gene both Reactome and 20x fold change

References

1. Periasamy, M.; Reed, T.D.; Liu, L.H.; Ji, Y.; Loukianov, E.; Paul, R.J.; Nieman, M.L.; Riddle, T.; Duffy, J.J.; Doetschman, T.; et al. Impaired Cardiac Performance in Heterozygous Mice with a Null Mutation in the Sarco(Endo)Plasmic Reticulum Ca2+-ATPase Isoform 2 (SERCA2) Gene. *J. Biol. Chem.* **1999**, *274*, 2556–2562, doi:10.1074/jbc.274.4.2556.
2. Pan, Y.; Zvaritch, E.; Tupling, A.R.; Rice, W.J.; de Leon, S.; Rudnicki, M.; McKerlie, C.; Banwell, B.L.; MacLennan, D.H. Targeted Disruption of the ATP2A1 Gene Encoding the Sarco(Endo)Plasmic Reticulum Ca2+ ATPase Isoform 1 (SERCA1) Impairs Diaphragm Function and Is Lethal in Neonatal Mice. *Journal of Biological Chemistry* **2003**, *278*, 13367–13375, doi:10.1074/jbc.M213228200.
3. Leva, F.; Domi, T.; Fedrizzi, L.; Lim, D.; Carafoli, E. The Plasma Membrane Ca2+ ATPase of Animal Cells: Structure, Function and Regulation. *Archives of Biochemistry and Biophysics* **2008**, *476*, 65–74, doi:10.1016/j.abb.2008.02.026.
4. Jones, H.D.; Li, T.Y.; Arystarkhova, E.; Barr, K.J.; Wetzel, R.K.; Peng, J.; Markham, K.; Sweadner, K.J.; Fong, G.-H.; Kidder, G.M. Na,K-ATPase from Mice Lacking the γ Subunit (FXYD2) Exhibits Altered Na+ Affinity and Decreased Thermal Stability. *Journal of Biological Chemistry* **2005**, *280*, 19003–19011, doi:10.1074/jbc.M500697200.
5. Magyar, J.P.; Bartsch, U.; Wang, Z.Q.; Howells, N.; Aguzzi, A.; Wagner, E.F.; Schachner, M. Degeneration of Neural Cells in the Central Nervous System of Mice Deficient in the Gene for the Adhesion Molecule on Glia, the B2 Subunit of Murine Na,K-ATPase. *J. Cell Biol.* **1994**, *127*, 835–845, doi:10.1083/jcb.127.3.835.
6. Jimenez, T.; McDermott, J.P.; Sánchez, G.; Blanco, G. Na,K-ATPase A4 Isoform Is Essential for Sperm Fertility. *PNAS* **2011**, *108*, 644–649, doi:10.1073/pnas.1016902108.
7. Kato, U.; Inadome, H.; Yamamoto, M.; Emoto, K.; Kobayashi, T.; Umeda, M. Role for Phospholipid Flippase Complex of ATP8A1 and CDC50A Proteins in Cell Migration. *Journal of Biological Chemistry* **2013**, *288*, 4922–4934, doi:10.1074/jbc.M112.402701.
8. Weingarten, L.S.; Dave, H.; Li, H.; Crawford, D.A. Developmental Expression of P5 ATPase MRNA in the Mouse. *Cellular and Molecular Biology Letters* **2012**, *17*, 153–170, doi:10.2478/s11658-011-0039-3.
9. Billig, G.M.; Pál, B.; Fidzinski, P.; Jentsch, T.J. Ca2+-Activated Cl− Currents Are Dispensable for Olfaction. *Nature Neuroscience* **2011**, *14*, 763–769, doi:10.1038/nn.2821.
10. Duran, C.; Qu, Z.; Osunkoya, A.O.; Cui, Y.; Criss Hartzell, H. ANOs 3-7 in the Anoctamin/Tmem16 Cl - Channel Family Are Intracellular Proteins. *American Journal of Physiology - Cell Physiology* **2012**, *302*, 482–493, doi:10.1152/ajpcell.00140.2011.
11. Krämer, F.; Stöhr, H.; Weber, B.H.F. Cloning and Characterization of the Murine Vmd2 RFP-TM Gene Family. *Cytogenet Genome Res* **2004**, *105*, 107–114, doi:10.1159/000078016.
12. Gyobu, S.; Miyata, H.; Ikawa, M.; Yamazaki, D.; Takeshima, H.; Suzuki, J.; Nagata, S. A Role of TMEM16E Carrying a Scrambling Domain in Sperm Motility. *Molecular and Cellular Biology* **2016**, *36*, 645–659, doi:10.1128/MCB.00919-15.
13. Piwon, N.; Gunther, W.; Schwake, M.; Bosl, M.R.; Jentsch, T.J. ClC-5 Cl- -Channel Disruption Impairs Endocytosis in a Mouse Model for Dent's Disease. *Nature* **2000**, *408*, 369–373, doi:10.1038/35042597.

14. Gronemeier, M.; Condie, A.; Prosser, J.; Steinmeyer, K.; Jentsch, T.J.; Jockusch, H. Nonsense and Missense Mutations in the Muscular Chloride Channel Gene Clc-1 of Myotonic Mice. *Journal of Biological Chemistry* **1994**, *269*, 5963–5967.
15. Wang, Y.; Fehlhaber, K.E.; Sarria, I.; Cao, Y.; Ingram, N.T.; Guerrero-Given, D.; Throesch, B.; Baldwin, K.; Kamasawa, N.; Ohtsuka, T.; et al. The Auxiliary Calcium Channel Subunit A2δ4 Is Required for Axonal Elaboration, Synaptic Transmission, and Wiring of Rod Photoreceptors. *Neuron* **2017**, *93*, 1359-1374.e6, doi:10.1016/j.neuron.2017.02.021.
16. Freise, D.; Held, B.; Wissenbach, U.; Pfeifer, A.; Trost, C.; Himmerkus, N.; Schweig, U.; Freichel, M.; Biel, M.; Hofmann, F.; et al. Absence of the γ Subunit of the Skeletal Muscle Dihydropyridine Receptor Increases L-Type Ca2+ Currents and Alters Channel Inactivation Properties. *Journal of Biological Chemistry* **2000**, *275*, 14476–14481.
17. Arikkath, J.; Campbell, K. Auxiliary Subunits: Essential Components of the Voltage-Gated Calcium Channel Complex. *Curr Opin Neurobiol* **2003**, *13*, 298–307, doi:10.1016/S0959-4388(03)00066-7.
18. Burgess, D.L.; Gefrides, L.A.; Foreman, P.J.; Noebels, J.L. A Cluster of Three Novel Ca2+ Channel γ Subunit Genes on Chromosome 19q13.4: Evolution and Expression Profile of the γ Subunit Gene Family. *Genomics* **2001**, *71*, 339–350, doi:10.1006/geno.2000.6440.
19. Jonker, J.W.; Wagenaar, E.; van Eijl, S.; Schinkel, A.H. Deficiency in the Organic Cation Transporters 1 and 2 (Oct1/Oct2 [Slc22a1/Slc22a2]) in Mice Abolishes Renal Secretion of Organic Cations. *Mol Cell Biol* **2003**, *23*, 7902–7908, doi:10.1128/mcb.23.21.7902-7908.2003.
20. Gawenis, L.R.; Greeb, J.M.; Prasad, V.; Grisham, C.; Sanford, L.P.; Doetschman, T.; Andringa, A.; Miller, M.L.; Shull, G.E. Impaired Gastric Acid Secretion in Mice with a Targeted Disruption of the NHE4 Na+/H+ Exchanger. *Journal of Biological Chemistry* **2005**, *280*, 12781–12789, doi:10.1074/jbc.M414118200.
21. Palmieri, F. The Mitochondrial Transporter Family SLC25: Identification, Properties and Physiopathology. *Mol Aspects Med* **2013**, *34*, 465–484, doi:10.1016/j.mam.2012.05.005.
22. Hentschke, M.; Wiemann, M.; Hentschke, S.; Kurth, I.; Hermans-Borgmeyer, I.; Seidenbecher, T.; Jentsch, T.J.; Gal, A.; Hübner, C.A. Mice with a Targeted Disruption of the Cl-/HCO3- Exchanger AE3 Display a Reduced Seizure Threshold. *Mol. Cell. Biol.* **2006**, *26*, 182–191, doi:10.1128/MCB.26.1.182-191.2006.
23. Jeon, D.; Yang, Y.-M.; Jeong, M.-J.; Philipson, K.D.; Rhim, H.; Shin, H.-S. Enhanced Learning and Memory in Mice Lacking Na+/Ca2+ Exchanger 2. *Neuron* **2003**, *38*, 965–976, doi:10.1016/S0896-6273(03)00334-9.
24. Sinasac, D.S.; Moriyama, M.; Jalil, M.A.; Begum, L.; Li, M.X.; Iijima, M.; Horiuchi, M.; Robinson, B.H.; Kobayashi, K.; Saheki, T.; et al. Slc25a13-Knockout Mice Harbor Metabolic Deficits but Fail To Display Hallmarks of Adult-Onset Type II Citrullinemia. *Molecular and Cellular Biology* **2004**, *24*, 527–536, doi:10.1128/mcb.24.2.527-536.2004.
25. Jacobs, S.; Ruusuvuori, E.; Sipilä, S.T.; Haapanen, A.; Damkier, H.H.; Kurth, I.; Hentschke, M.; Schweizer, M.; Rudhard, Y.; Laatikainen, L.M.; et al. Mice with Targeted Slc4a10 Gene Disruption Have Small Brain Ventricles and Show Reduced Neuronal Excitability. *PNAS* **2008**, *105*, 311–316, doi:10.1073/pnas.0705487105.
26. Wojcik, S.M.; Rhee, J.S.; Herzog, E.; Sigler, A.; Jahn, R.; Takamori, S.; Brose, N.; Rosenmund, C. An Essential Role for Vesicular Glutamate Transporter 1 (VGLUT1) in Postnatal Development and Control of Quantal Size. *PNAS* **2004**, *101*, 7158–7163, doi:10.1073/pnas.0401764101.
27. Mariotta, L.; Ramadan, T.; Singer, D.; Guetg, A.; Herzog, B.; Stoeger, C.; Palacín, M.; Lahoutte, T.; Camargo, S.M.R.; Verrey, F. T-type Amino Acid Transporter TAT1 (Slc16a10) Is Essential for Extracellular Aromatic Amino Acid Homeostasis Control. *J Physiol* **2012**, *590*, 6413–6424, doi:10.1113/jphysiol.2012.239574.
28. Braun, D.; Wirth, E.K.; Wohlgemuth, F.; Reix, N.; Klein, M.O.; Grüters, A.; Köhrle, J.; Schweizer, U. Aminoaciduria, but Normal Thyroid Hormone Levels and Signalling, in Mice Lacking the Amino Acid and Thyroid Hormone Transporter Slc7a8. *Biochemical Journal* **2011**, *439*, 249–255, doi:10.1042/BJ20110759.
29. Deisl, C.; Simonin, A.; Anderegg, M.; Albano, G.; Kovacs, G.; Ackermann, D.; Moch, H.; Dolci, W.; Thorens, B.; Hediger, M.A.; et al. Sodium/Hydrogen Exchanger NHA2 Is Critical for Insulin Secretion in β-Cells. *PNAS* **2013**, *110*, 10004–10009, doi:10.1073/pnas.1220009110.
30. Gawenis, L.R.; Bradford, E.M.; Prasad, V.; Lorenz, J.N.; Simpson, J.E.; Clarke, L.L.; Woo, A.L.; Grisham, C.; Sanford, L.P.; Doetschman, T.; et al. Colonic Anion Secretory Defects and Metabolic Acidosis in Mice Lacking the NBC1 Na+/HCO3- Cotransporter. *Journal of Biological Chemistry* **2006**, *282*, 9042–9052, doi:10.1074/jbc.M607041200.

31. Sokolow, S.; Manto, M.; Gailly, P.; Molgó, J.; Vandebrouck, C.; Vanderwinden, J.-M.M.; Herchuelz, A.; Schurmans, S. Impaired Neuromuscular Transmission and Skeletal Muscle Fiber Necrosis in Mice Lacking Na/Ca Exchanger 3. *Journal of Clinical Investigation* **2004**, *113*, 265–273, doi:10.1172/JCI18688.
32. Cho, C.-H.; Kim, S.S.; Jeong, M.; Lee, C.O.; Shin, H.-S. The Na+ -Ca2+ Exchanger Is Essential for Embryonic Heart Development in Mice. *Mol. Cells* **2000**, *10*, 712–722, doi:10.1007/s10059-000-0712-2.
33. Wakimoto, K.; Kobayashi, K.; Kuro-o, M.; Yao, A.; Iwamoto, T.; Yanaka, N.; Kita, S.; Nishida, A.; Azuma, S.; Toyoda, Y.; et al. Targeted Disruption of Na + /Ca 2+ Exchanger Gene Leads to Cardiomyocyte Apoptosis and Defects in Heartbeat. *Journal of Biological Chemistry* **2000**, *275*, 36991–36998, doi:10.1074/jbc.M004035200.
34. Chintala, S.; Tan, J.; Gautam, R.; Rusiniak, M.E.; Guo, X.; Li, W.; Gahl, W.A.; Huizing, M.; Spritz, R.A.; Hutton, S.; et al. The Slc35d3 Gene, Encoding an Orphan Nucleotide Sugar Transporter, Regulates Platelet-Dense Granules. *Blood* **2007**, *109*, 1533–1540, doi:10.1182/blood-2006-08-040196.
35. Tokuda, N.; Numata, S.; Li, X.; Nomura, T.; Takizawa, M.; Kondo, Y.; Yamashita, Y.; Hashimoto, N.; Kiyono, T.; Urano, T.; et al. B4GalT6 Is Involved in the Synthesis of Lactosylceramide with Less Intensity than B4GalT5. *Glycobiology* **2013**, *23*, 1175–1183, doi:10.1093/glycob/cwt054.
36. Tomatsu, S.; Orii, K.O.; Vogler, C.; Nakayama, J.; Levy, B.; Grubb, J.H.; Gutierrez, M.A.; Shim, S.; Yamaguchi, S.; Nishioka, T.; et al. Mouse Model on N-Acetylgalactosamine-6-Sulfate Sulfatase Deficiency (Galns-/-) Produced by Targeted Disruption of the Gene Defective in Morquio A Disease. *Human Molecular Genetics* **2003**, *12*, 3349–3358, doi:10.1093/hmg/ddg366.
37. Yamamoto, S.; Oka, S.; Inoue, M.; Shimuta, M.; Manabe, T.; Takahashi, H.; Miyamoto, M.; Asano, M.; Sakagami, J.; Sudo, K.; et al. Mice Deficient in Nervous System-Specific Carbohydrate Epitope HNK-1 Exhibit Impaired Synaptic Plasticity and Spatial Learning. *Journal of Biological Chemistry* **2002**, *277*, 27227–27231, doi:10.1074/jbc.C200296200.
38. Yang, W.H.; Nussbaum, C.; Grewal, P.K.; Marth, J.D.; Sperandio, M. Coordinated Roles of ST3Gal-VI and ST3Gal-IV Sialyltransferases in the Synthesis of Selectin Ligands. *Blood* **2012**, *120*, 1015–1026, doi:10.1182/blood-2012-04-424366.
39. Babu, G.J.; Bhupathy, P.; Timofeyev, V.; Petrashevskaya, N.N.; Reiser, P.J.; Chiamvimonvat, N.; Periasamy, M. Ablation of Sarcolipin Enhances Sarcoplasmic Reticulum Calcium Transport and Atrial Contractility. *PNAS* **2007**, *104*, 17867–17872.
40. Butz, S.; Fernandez-Chacon, R.; Schmitz, F.; Jahn, R.; Südhof, T.C. The Subcellular Localizations of Atypical Synaptotagmins III and VI. Synaptotagmin III Is Enriched in Synapses and Synaptic Plasma Membranes but Not in Synaptic Vesicles. *Journal of Biological Chemistry* **1999**, *274*, 18290–18296, doi:10.1074/jbc.274.26.18290.
41. Yagi, H.; Takamura, Y.; Yoneda, T.; Konno, D.; Akagi, Y.; Yoshida, K.; Sato, M. Vlgr1 Knockout Mice Show Audiogenic Seizure Susceptibility. *Journal of Neurochemistry* **2005**, *92*, 191–202, doi:10.1111/j.1471-4159.2004.02875.x.
42. Knollmann, B.C.; Chopra, N.; Hlaing, T.; Akin, B.; Yang, T.; Ettensohn, K.; Knollmann, B.E.C.; Horton, K.D.; Weissman, N.J.; Holinstat, I.; et al. Casq2 Deletion Causes Sarcoplasmic Reticulum Volume Increase, Premature Ca2+ Release, and Catecholaminergic Polymorphic Ventricular Tachycardia. *Journal of Clinical Investigation* **2006**, *116*, 2510–2520, doi:10.1172/JCI29128.
43. Gyurko, R.; Leupen, S.; Huang, P.L. Deletion of Exon 6 of the Neuronal Nitric Oxide Synthase Gene in Mice Results in Hypogonadism and Infertility. *Endocrinology* **2002**, *143*, 2767–2774.
44. Silva, A.J.; Stevens, C.F.; Tonegawa, S.; Wang, Y. Deficient Hippocampal Long-Term Potentiation in Alpha-Calcium-Calmodulin Kinase II Mutant Mice. *Science* **1992**, *257*, 201–206, doi:10.1126/science.1378648.
45. Paolini, C.; Quarta, M.; Nori, A.; Boncompagni, S.; Canato, M.; Volpe, P.; Allen, P.D.; Reggiani, C.; Protasi, F. Reorganized Stores and Impaired Calcium Handling in Skeletal Muscle of Mice Lacking Calsequestrin-1. *Journal of Physiology* **2007**, *583*, 767–784, doi:10.1113/jphysiol.2007.138024.
46. Suzuki, M.; Mizuno, A.; Kodaira, K.; Imai, M. Impaired Pressure Sensation in Mice Lacking TRPV4. *Journal of Biological Chemistry* **2003**, *278*, 22664–22668, doi:10.1074/jbc.M302561200.
47. Koike, C.; Obara, T.; Uriu, Y.; Numata, T.; Sanuki, R.; Miyata, K.; Koyasu, T.; Ueno, S.; Funabiki, K.; Tani, A.; et al. TRPM1 Is a Component of the Retinal ON Bipolar Cell Transduction Channel in the MGluR6 Cascade. *PNAS* **2010**, *107*, 332–337, doi:10.1073/pnas.0912730107.
48. Hummler, E.; Barker, P.; Gatzy, J.; Beermann, F.; Verdumo, C.; Schmidt, A.; Boucher, R.; Rossier, B.C. Early Death Due to Defective Neonatal Lung Liquid Clearance in Alpha-ENaC-Deficient Mice. *Nature Genetics* **1996**, *12*, 325–328.

49. Nassar, M.A.; Baker, M.D.; Levato, A.; Ingram, R.; Mallucci, G.; McMahon, S.B.; Wood, J.N. Nerve Injury Induces Robust Allodynia and Ectopic Discharges in Nav 1.3 Null Mutant Mice. *Molecular Pain* **2006**, *2*, 1–10, doi:10.1186/1744-8069-2-33.
50. Hayward, L.J.; Kim, J.S.; Lee, M.-Y.; Zhou, H.; Kim, J.W.; Misra, K.; Salajegheh, M.; Wu, F.; Matsuda, C.; Reid, V.; et al. Targeted Mutation of Mouse Skeletal Muscle Sodium Channel Produces Myotonia and Potassium-Sensitive Weakness. *Journal of Clinical Investigation* **2008**, *118*, 1437–1449, doi:10.1172/JCI32638.
51. GARNIER, G.; CIRCOLO, A.; XU, Y.; VOLANAKIS, J.E. Complement C1r and C1s Genes Are Duplicated in the Mouse: Differential Expression Generates Alternative Isomorphs in the Liver and in the Male Reproductive System. *Biochem J* **2003**, *371*, 631–640.
52. Nieuwenhuis, E.E.S.; Neurath, M.F.; Corazza, N.; Iijima, H.; Trgovcich, J.; Wirtz, S.; Glickman, J.; Bailey, D.; Yoshida, M.; Galle, P.R.; et al. Disruption of T Helper 2-Immune Responses in Epstein-Barr Virus-Induced Gene 3-Deficient Mice. *Proceedings of the National Academy of Sciences of the United States of America* **2002**, *99*, 16951–16956, doi:10.1073/pnas.252648899.
53. Sarter, K.; Leimgruber, E.; Gobet, F.; Agrawal, V.; Dunand-Sauthier, I.; Barras, E.; Mastelic-Gavillet, B.; Kamath, A.; Fontannaz, P.; Guéry, L.; et al. Btn2a2, a T Cell Immunomodulatory Molecule Coregulated with MHC Class II Genes. *Journal of Experimental Medicine* **2016**, *213*, 177–187, doi:10.1084/jem.20150435.
54. Kallio, H.; Tolvanen, M.; Jänis, J.; Pan, P.; Laurila, E.; Kallioniemi, A.; Kilpinen, S.; Tuominen, V.J.; Isola, J.; Valjakka, J.; et al. Characterization of Non-Specific Cytotoxic Cell Receptor Protein 1: A New Member of the Lectin-Type Subfamily of F-Box Proteins. *PLoS ONE* **2011**, *6*, e27152, doi:10.1371/journal.pone.0027152.
55. Lei, H.; Sun, Y.; Luo, Z.; Yourek, G.; Gui, H.; Yang, Y.; Su, D.F.; Liu, X. Fatigue-Induced Orosomucoid 1 Acts on C-C Chemokine Receptor Type 5 to Enhance Muscle Endurance. *Scientific Reports* **2016**, *6*, 1–9, doi:10.1038/srep18839.
56. McQueen, K.L.; Freeman, J.D.; Takei, F.; Mager, D.L. Localization of Five New Ly49 Genes, Including Three Closely Related to Ly49c. *Immunogenetics* **1998**, *48*, 174–183, doi:10.1007/s002510050421.
57. Johansen, F.-E.; Pekna, M.; Norderhaug, I.N.; Haneberg, B.; Hietala, M.A.; Krajci, P.; Betsholtz, C.; Brandtzaeg, P. Absence of Epithelial Immunoglobulin A Transport, with Increased Mucosal Leakiness, in Polymeric Immunoglobulin Receptor/Secretory Component-Deficient Mice. *J. Exp. Med.* **1999**, *190*, 915–922, doi:10.1084/jem.190.7.915.
58. Rosenzweig, M. Identification of VSIG8 as the Putative VISTA Receptor and Its Use Thereof to Produce VISTA/VSIG8 Modulators 2009.
59. Winkler, M.; Biswas, S.; Berger, S.M.; Küchler, M.; Preisendörfer, L.; Choo, M.; Früh, S.; Rem, P.D.; Enkel, T.; Arnold, B.; et al. Pianp Deficiency Links GABAB Receptor Signaling and Hippocampal and Cerebellar Neuronal Cell Composition to Autism-like Behavior. *Molecular Psychiatry* **2019**, 1–15, doi:10.1038/s41380-019-0519-9.
60. Washington, A.V.; Gibot, S.; Acevedo, I.; Gattis, J.; Quigley, L.; Feltz, R.; de La Mota, A.; Schubert, R.L.; Gomez-Rodriguez, J.; Cheng, J.; et al. TREM-like Transcript-1 Protects against Inflammation-Associated Hemorrhage by Facilitating Platelet Aggregation in Mice and Humans. *J Clin Invest* **2009**, *119*, 1489–1501, doi:10.1172/JCI36175.
61. Zafirova, B.; Mandarić, S.; Antulov, R.; Krmpotić, A.; Jonsson, H.; Yokoyama, W.M.; Jonjić, S.; Polić, B. Altered NK Cell Development and Enhanced NK Cell-Mediated Resistance to Mouse Cytomegalovirus in NKG2D-Deficient Mice. *Immunity* **2009**, *31*, 270–282, doi:10.1016/j.immuni.2009.06.017.
62. Waterhouse, P.; Penninger, J.M. Lymphoproliferative Disorders with Early Lethality in Mice Deficient in Ctla-4. *Science* **1995**, *270*, 985–988.
63. Sullivan, L.; Berry, R.; Sosnin, N.; Widjaja, J.; Deuss, F.; Balaji, G.; Nicole, L.; Mirams, M.; Trapani, J.; Rossjohn, J.; et al. Recognition of the Major Histocompatibility Complex (MHC) Class Ib Molecule H2-Q10 by the Natural Killer Cell Receptor Ly49C. *J Biol Chem* **2016**, *291*, 18740–18752, doi:10.1074/jbc.M116.737130.
64. Orr, M.T.; Wu, J.; Fang, M.; Sigal, L.J.; Spee, P.; Egebjerg, T.; Dissen, E.; Fossum, S.; Phillips, J.H.; Lanier, L.L. Development and Function of CD94-Deficient Natural Killer Cells. *PLoS ONE* **2010**, *5*, doi:10.1371/journal.pone.0015184.
65. Clauss, A.; Lilja, H.; Lundwall, Å. The Evolution of a Genetic Locus Encoding Small Serine Proteinase Inhibitors. *Biochemical and Biophysical Research Communications* **2005**, *333*, 383–389, doi:10.1016/j.bbrc.2005.05.125.

66. Uematsu, S.; Jang, M.H.; Chevrier, N.; Guo, Z.; Kumagai, Y.; Yamamoto, M.; Kato, H.; Sougawa, N.; Matsui, H.; Kuwata, H.; et al. Detection of Pathogenic Intestinal Bacteria by Toll-like Receptor 5 on Intestinal CD11c+ Lamina Propria Cells. *Nature Immunology* **2006**, *7*, 868–874, doi:10.1038/ni1362.
67. Eura, Y.; Yanamoto, H.; Arai, Y.; Okuda, T.; Miyata, T.; Kokame, K. Derlin-1 Deficiency Is Embryonic Lethal, Derlin-3 Deficiency Appears Normal, and Herp Deficiency Is Intolerant to Glucose Load and Ischemia in Mice. *PLoS ONE* **2012**, *7*, e34298, doi:10.1371/journal.pone.0034298.
68. Elamaa, H.; Kihlström, M.; Kapiainen, E.; Kaakinen, M.; Miinalainen, I.; Ragauskas, S.; Cerrada-Gimenez, M.; Mering, S.; Nätynki, M.; Eklund, L.; et al. Angiopoietin-4-Dependent Venous Maturation and Fluid Drainage in the Peripheral Retina. *eLife* **2018**, *7*, 1–32, doi:10.7554/eLife.37776.
69. Aoki, M.; Mieda, M.; Ikeda, T.; Hamada, Y.; Nakamura, H.; Okamoto, H. R-Spondin3 Is Required for Mouse Placental Development. *Developmental Biology* **2007**, *301*, 218–226, doi:10.1016/j.ydbio.2006.08.018.
70. Howell, B.W.; Hawkes, R.; Soriano, P.; Cooper, J.A. Neuronal Position in the Developing Brain Is Regulated by Mouse Disabled-1. *Nature* **1997**, *389*, 733–737, doi:10.1038/39607.
71. Taniguchi, T.; Tanaka, S.; Ishii, A.; Watanabe, M.; Fujitani, N.; Sugeo, A.; Gotoh, S.; Ohta, T.; Hiyoshi, M.; Matsuzaki, H.; et al. A Brain-Specific Grb2-Associated Regulator of Extracellular Signal-Regulated Kinase (Erk)/Mitogen-Activated Protein Kinase (MAPK) (GAREM) Subtype, GAREM2, Contributes to Neurite Outgrowth of Neuroblastoma Cells by Regulating Erk Signaling. *J Biol Chem* **2013**, *288*, 29934–29942, doi:10.1074/jbc.M113.492520.
72. Jun, K.; Choi, G.; Yang, S.; Choi, K.Y.; Kim, H.; Chan, G.C.K.; Storm, D.R.; Albert, C.; Mayr, G.W.; Lee, C.; et al. Enhanced Hippocampal CA1 LTP but Normal Spatial Learning 3-Kinase (A) -Deficient Mice. *Learning & Memory* **1998**, *5*, 317–330.
73. Ho, C.; Conner, D.A.; Pollak, M.R.; Ladd, D.J.; Kifor, O.; Warren, H.B.; Brown, E.M.; Seidman, J.G.; Seidman, C.E. A Mouse Model of Human Familial Hypocalciuric Hypercalcemia and Neonatal Severe Hyperparathyroidism. *Nature Genetics* **1995**, *11*, 389–394, doi:10.1038/ng1295-389.
74. Schwaller, B.; Dick, J.; Dhoot, G.; Carroll, S.; Vrbova, G.; Nicotera, P.; Pette, D.; Wyss, A.; Bluethmann, H.; Hunziker, W.; et al. Prolonged Contraction-Relaxation Cycle of Fast-Twitch Muscles in Parvalbumin Knockout Mice. *American Journal of Physiology - Cell Physiology* **1999**, *276*, 395–403, doi:10.1152/ajpcell.1999.276.2.c395.
75. Leclerc, E.A.; Huchenq, A.; Kezic, S.; Serre, G.; Jonca, N. Mice Deficient for the Epidermal Dermokine β and γ Isoforms Display Transient Cornification Defects. *J Cell Sci* **2014**, *127*, 2862–2872, doi:10.1242/jcs.144808.
76. Morgan, D.J.; Wei, S.; Gomes, I.; Czyzyk, T.; Mzhavia, N.; Pan, H.; Devi, L.A.; Fricker, L.D.; Pintar, J.E. The Propeptide Precursor ProSAAS Is Involved in Fetal Neuropeptide Processing and Body Weight Regulation. *Journal of Neurochemistry* **2010**, *113*, 1275–1284, doi:10.1111/j.1471-4159.2010.06706.x.
77. Hellström, A.R.; Watt, B.; Fard, S.S.; Tenza, D.; Mannström, P.; Narfström, K.; Ekesten, B.; Ito, S.; Wakamatsu, K.; Larsson, J.; et al. Inactivation of Pmel Alters Melanosome Shape But Has Only a Subtle Effect on Visible Pigmentation. *PLoS Genetics* **2011**, *7*, e1002285, doi:10.1371/journal.pgen.1002285.
78. Zhang, X.; Firestein, S. The Olfactory Receptor Gene Superfamily of the Mouse. *Nature Neuroscience* **2002**, *5*, 124–133, doi:10.1038/nn800.
79. Asai, M.; Ramachandrappa, S.; Joachim, M.; Shen, Y.; Zhang, R.; Nuthalapati, N.; Ramanathan, V.; Strochlic, D.E.; Ferket, P.; Linhart, K.; et al. Loss of Function of the Melanocortin 2 Receptor Accessory Protein 2 Is Associated with Mammalian Obesity. *Science* **2013**, *341*, 275–278, doi:10.1126/science.1233000.
80. Miyawaki, K.; Yamada, Y.; Yano, H.; Niwa, H.; Ban, N.; Ihara, Y.; Kubota, A.; Fujimoto, S.; Kajikawa, M.; Kuroe, A.; et al. Glucose Intolerance Caused by a Defect in the Entero-Insular Axis: A Study in Gastric Inhibitory Polypeptide Receptor Knockout Mice. *Proceedings of the National Academy of Sciences* **1999**, *96*, 14843–14847, doi:10.1073/pnas.96.26.14843.
81. Sun, Y.; Ahmed, S.; Smith, R.G. Deletion of Ghrelin Impairs Neither Growth nor Appetite. *Molecular and Cellular Biology* **2003**, *23*, 7973–7981, doi:10.1128/mcb.23.22.7973-7981.2003.
82. Hansen, T. v; Hammer, N.A.; Nielsen, J.; Madsen, M.; Dalbaeck, C.; Wewer, U.M.; Christiansen, J.; Nielsen, F.C. Dwarfism and Impaired Gut Development in Insulin-like Growth Factor II MRNA-Binding Protein 1-Deficient Mice. *Mol. Cell. Biol.* **2004**, *24*, 4448–4464, doi:10.1128/mcb.24.10.4448-4464.2004.
83. Champy, M.-F.; Voci, L.; Selloum, M.; Peterson, L.; Cumiskey, A.; Blom, D. Reduced Body Weight in Male Tspan8-Deficient Mice. *Int J Obesity* **2011**, *35*, 605–617, doi:10.1038/ijo.2010.165.
84. Schmit, K.; Michiels, C. TMEM Proteins in Cancer: A Review. *Front Pharmacol* **2018**, *9*, 1345, doi:10.3389/fphar.2018.01345.

85. McPherron, a C.; Lawler, a M.; Lee, S.J. Regulation of Skeletal Muscle Mass in Mice by a New TGF-Beta Superfamily Member [In Process Citation]. *Nature* **1997**, *387*, 83–90.
86. Zhang, J.; Zhang, X.; Zhang, Y.; Zeng, W.; Zhao, S.; Liu, M. Normal Spermatogenesis in Fank1 (Fibronectin Type 3 and Ankyrin Repeat Domains 1) Mutant Mice. *PeerJ* **2019**, *7*, e6827, doi:10.7717/peerj.6827.
87. Ehrlich, K.C.; Lacey, M.; Ehrlich, M. Epigenetics of Skeletal Muscle-Associated Genes in the ASB, LRRC, TMEM, and OSBPL Gene Families. *Epigenomes* **2020**, *4*, 1, doi:10.3390/epigenomes4010001.
88. Huang, L.; Mivechi, N.F.; Moskophidis, D. Insights into Regulation and Function of the Major Stress-Induced Hsp70 Molecular Chaperone in Vivo: Analysis of Mice with Targeted Gene Disruption of the Hsp70.1 or Hsp70.3 Gene. *Molecular and Cellular Biology* **2001**, *21*, 8575–8591.
89. Shaw, G. The Pleckstrin Homology Domain: An Intriguing Multifunctional Protein Module. *Bioessays* **1996**, *18*, 35–46, doi:10.1002/bies.950180109.
90. Collins, T.; Stone, J.R.; Williams, A.J. All in the Family: The BTB/POZ, KRAB, and SCAN Domains. *Mol. Cell. Biol.* **2001**, *21*, 3609–3615, doi:10.1128/MCB.21.11.3609-3615.2001.
91. Pei, J.; Grishin, N. Unexpected Diversity in Shisa-like Proteins Suggests the Importance of Their Roles as Transmembrane Adaptors. *Cell Signal* **2012**, *24*, 758–769, doi:10.1016/j.cellsig.2011.11.011.
92. Zhang, Y.; Deng, W.T.; Du, W.; Zhu, P.; Li, J.; Xu, F.; Sun, J.; Gerstner, C.D.; Baehr, W.; Sanford, L.B.; et al. Gene-Based Therapy in a Mouse Model of Blue Cone Monochromacy. *Scientific Reports* **2017**, *7*, 1–8, doi:10.1038/s41598-017-06982-7.
93. Gao, J.; Cheon, K.; Nusinowitz, S.; Liu, Q.; Bei, D.; Atkins, K.; Azimi, A.; Daiger, S.P.; Farber, D.B.; Heckenlively, J.R.; et al. Progressive Photoreceptor Degeneration, Outer Segment Dysplasia, and Rhodopsin Mislocalization in Mice with Targeted Disruption of the Retinitis Pigmentosa-1 (Rp1) Gene. *Proc. Natl. Acad. Sci. USA* **2002**, *99*, 5698–5703, doi:10.1073/pnas.042122399.
94. Capoano, C.A.; Wettstein, R.; Kun, A.; Geisinger, A. Spats 1 (Srsp1) Is Differentially Expressed during Testis Development of the Rat. *Gene Expr Patterns* **2010**, *10*, 1–8, doi:10.1016/j.gep.2009.11.006.
95. Ru, Y.-F.; Xue, H.-M.; Ni, Z.-M.; Xia, D.; Zhou, Y.-C.; Zhang, Y.-L. An Epididymis-Specific Carboxyl Esterase CES5A Is Required for Sperm Capacitation and Male Fertility in the Rat. *Asian J Androl* **2015**, *17*, 292–297, doi:10.4103/1008-682X.143314.
96. Zhang, Z.; Shen, X.; Gude, D.R.; Wilkinson, B.M.; Justice, M.J.; Flickinger, C.J.; Herr, J.C.; Eddy, E.M.; Strauss, J.F. MEIG1 Is Essential for Spermiogenesis in Mice. *Proceedings of the National Academy of Sciences* **2009**, *106*, 17055–17060, doi:10.1073/pnas.0906414106.
97. Miller, T.; Williams, K.; Johnstone, R.W.; Shilatifard, A. Identification, Cloning, Expression, and Biochemical Characterization of the Testis-Specific RNA Polymerase II Elongation Factor ELL3. *Journal of Biological Chemistry* **2000**, *275*, 32052–32056, doi:10.1074/jbc.M005175200.
98. Choi, E.; Cho, C. Expression of a Sperm Flagellum Component Encoded by the Als2cr12 Gene. *Gene Expression Patterns* **2011**, *11*, 327–333, doi:10.1016/j.gep.2011.03.003.
99. Rankin, T.L.; O'Brien, M.; Lee, E.; Wigglesworth, K.; Eppig, J.; Dean, J. Defective Zonae Pellucidae in Zp2-Null Mice Disrupt Folliculogenesis, Fertility and Development. *Development* **2001**, *128*, 1119–1126.
100. Fujino, A.; Pieretti-Vanmarcke, R.; Wong, A.; Donahoe, P.K.; Arango, N.A. Sexual Dimorphism of G-protein Subunit Gng13 Expression in the Cortical Region of the Developing Mouse Ovary. *Developmental Dynamics* **2007**, *236*, 1991–1996, doi:10.1002/dvdy.21183.
101. Toyoda, S.; Miyazaki, T.; Miyazaki, S.; Yoshimura, T.; Yamamoto, M.; Tashiro, F.; Yamato, E.; Miyazaki, J. ichi Sohlh2 Affects Differentiation of KIT Positive Oocytes and Spermatogonia. *Developmental Biology* **2009**, *325*, 238–248, doi:10.1016/j.ydbio.2008.10.019.
102. Chung, J.-J.; Navarro, B.; Krapivinsky, G.; Krapivinsky, L.; Clapham, D.E. A Novel Gene Required for Male Fertility and Functional CATSPER Channel Formation in Spermatozoa. *Nature Communications* **2011**, *2*, 153, doi:10.1038/ncomms1153.
103. Rijnkels, M. Multispecies Comparison of the Casein Gene Loci and Evolution of Casein Gene Family. *Journal of Mammary Gland Biology and Neoplasia* **2002**, *7*, 327–345, doi:10.1023/A:1022808918013.
104. Garcia, V.; Gilani, A.; Shkolnik, B.; Pandey, V.; Zhang, F.F.; Dakarapu, R.; Gandham, S.K.; Reddy, N.R.; Graves, J.P.; Gruzdev, A.; et al. 20-HETE Signals Through G-Protein–Coupled Receptor GPR75 (Gq) to Affect Vascular Function and Trigger Hypertension. *Circ Res* **2017**, *120*, 1776–1788, doi:10.1161/CIRCRESAHA.116.310525.

105. Pumford, N.R.; Martin, B.M.; Hinson, J.A. A Metabolite of Acetaminophen Covalently Binds to the 56 KDa Selenium Binding Protein. *Biochem. Biophys. Res. Commun.* **1992**, *182*, 1348–1355, doi:10.1016/0006-291x(92)91881-p.
106. Nishida, H.; Suzuki, T.; Ookawa, H.; Tomaru, Y.; Hayashizaki, Y. Comparative Analysis of Expression of Histone H2a Genes in Mouse. *BMC Genomics* **2005**, *6*, 108, doi:10.1186/1471-2164-6-108.
